# Inhibitory Activity and Mechanism Investigation of Hypericin as a Novel *α*-Glucosidase Inhibitor

**DOI:** 10.3390/molecules26154566

**Published:** 2021-07-28

**Authors:** Qi Dong, Na Hu, Huilan Yue, Honglun Wang

**Affiliations:** 1 CAS Key Laboratory of Tibetan Medicine Research, Northwest Institute of Plateau Biology, Xining 810008, China; qdong@nwipb.cas.cn (Q.D.); huna@nwipb.cas.cn (N.H.); hlyue@nwipb.cas.cn (H.Y.); 2 Qinghai Provincial Key Laboratory of Tibetan Medicine Research, Xining 810008, China; 3 University of Chinese Academy of Sciences, Beijing 100049, China

**Keywords:** hypericin, α-glucosidase inhibitor, mechanism

## Abstract

*α*-glucosidase is a major enzyme that is involved in starch digestion and type 2 diabetes mellitus. In this study, the inhibition of hypericin by α-glucosidase and its mechanism were firstly investigated using enzyme kinetics analysis, real-time interaction analysis between hypericin and *α*-glucosidase by surface plasmon resonance (SPR), and molecular docking simulation. The results showed that hypericin was a high potential reversible and competitive α-glucosidase inhibitor, with a maximum half inhibitory concentration (IC_50_) of 4.66 ± 0.27 mg/L. The binding affinities of hypericin with *α*-glucosidase were assessed using an SPR detection system, which indicated that these were strong and fast, with balances dissociation constant (KD) values of 6.56 × 10^−5^ M and exhibited a slow dissociation reaction. Analysis by molecular docking further revealed that hydrophobic forces are generated by interactions between hypericin and amino acid residues Arg-315 and Tyr-316. In addition, hydrogen bonding occurred between hypericin and *α*-glucosidase amino acid residues Lys-156, Ser-157, Gly-160, Ser-240, His-280, Asp-242, and Asp-307. The structure and micro-environment of α-glucosidase enzymes were altered, which led to a decrease in α-glucosidase activity. This research identified that hypericin, an anthracene ketone compound, could be a novel α-glucosidase inhibitor and further applied to the development of potential anti-diabetic drugs.

## 1. Introduction

Diabetes mellitus (DM) pertains to a range of metabolic disorders that are characterized by chronic hyperglycemia, coupled with insufficiencies and/or dysfunctional insulin secretion [1]. Approximately 451 million people around the world have been diagnosed with diabetes in 2017, and this is expected to increase to 693 million by 2045 [2]. Hyperglycemia is the most important index of all types of diabetes and can lead to various complications, such as cardiovascular disease, renal failure, neuropathy, lipid metabolism disorder, and others [3,4]. Epidemiological studies showed that postprandial hyperglycemia was an important factor leading to impaired glucose tolerance and development of type 2 DM. Postprandial hyperglycemia is a major factor contributing to diabetic macrovascular complications and microvascular complications. Regulating postprandial blood glucose plays an important role in preventing vascular complications [5]. Maintaining postprandial blood glucose levels in a normal range is one of the important ways to control blood glucose fluctuation, which prevents the occurrence of cardiovascular and cerebrovascular diseases, and reduces the mortality of cardiovascular and cerebrovascular diseases [6]. Therefore, controlling the blood sugar level is very important for diabetics [7,8].

α-glucosidase, as an important carbohydrate hydrolase, plays a key role in the process of transforming oligosaccharides and disaccharides into glucose. The monosaccharide produced can be absorbed by the small intestine, leading to an increase in blood glucose levels [9]. Therefore, α-glucosidase is considered to be the main target enzyme for the prevention and treatment of diabetes [10,11]. Considering the difficulty of obtaining purity α-glucosidase from mammals, yeast α-glucosidase due to its ease of access is often used as a model for researching potential α-glucosidase inhibitors (AGI) and inhibitory mechanism [12]. Yeast α-glucosidase was classified as a member of the retaining glycoside hydrolase family 13 that included many important digestive enzymes and the hydrolytic reactions that occurred by splitting of the bond between the anomeric carbon of the glucosyl residue and glucosidic oxygen [13,14].

α-glucosidase inhibitors (AGIs) are a class of drugs that have been used for the treatment of type 2 DM. At present, AGIs are used as first-line drugs for the treatment of type 2 DM patients with poor diet control as well as the preferred adjuvant for type 1 DM patients receiving insulin therapy [15]. AGIs can reduce postprandial glucose levels by preventing the hydrolysis of polysaccharides to glucose and related monosaccharides, which then decreases the absorption of carbohydrates in the digestive tract [16]. At present, there are few varieties of *α*-glucosidase inhibitors, which mainly include acarbose, voglibose, and miglitol. However, the preparation process of these drugs is cumbersome and costly, and research progress on their synthesis is slow. Therefore, researchers prefer to screen new *α*-glucosidase inhibitors from natural product resources to identify safer and more effective drugs. Flavonoids, alkaloids, polysaccharides, and phenols have been shown to exhibit good *α*-glucosidase inhibitory activity [17].

*Hypericum perforatum* L. has been extensively utilized for its antibacterial, neuroprotective, antidepressant, antioxidant, menopause, dentistry, anti-inflammatory, wound healing, anticancer, antidepressant, and phototoxicity effects [18,19]. It also imparts hypoglycemic effects on diabetic rats induced by streptozotocin [20,21] and thus is a promising resource for the treatment of type 2DM [22]. We have studied the extract of *H. perforatum* L. and found that it exhibits strong *α*-glucosidase inhibitory activity. Hypericin (4,5,7,4′,5′,7′-hexahydroxy-2′,2′-dimethyl-o-naphthalene dione) is one of the main active components that consists of a hard polycyclic aromatic quinone. It was extracted from *H. perforatum* and showed significant pharmacological effects such as antiviral [23,24], antitumor [25,26], antidepressant [27,28,29], and antibacterial [30,31,32] effects. However, the inhibitory effect of hypericin, as well as its mechanism and kinetics on α-glucosidase remain unclear.

This study firstly assessed the *α*-glucosidase inhibitory activity of hypericin using a p-nitrophenyl-α-D-glucopyranoside (pNPG) substrate. In addition, the mechanism of hypericin on α-glucosidase was investigated by enzyme dynamics and real-time interaction analyses between hypericin and *α*-glucosidase using surface plasmon resonance (SPR) and molecular docking.

## 2. Results and Discussion

### 2.1. Inhibition of α-Glucosidase by Hypericin and Acarbose

**Figure 1:**
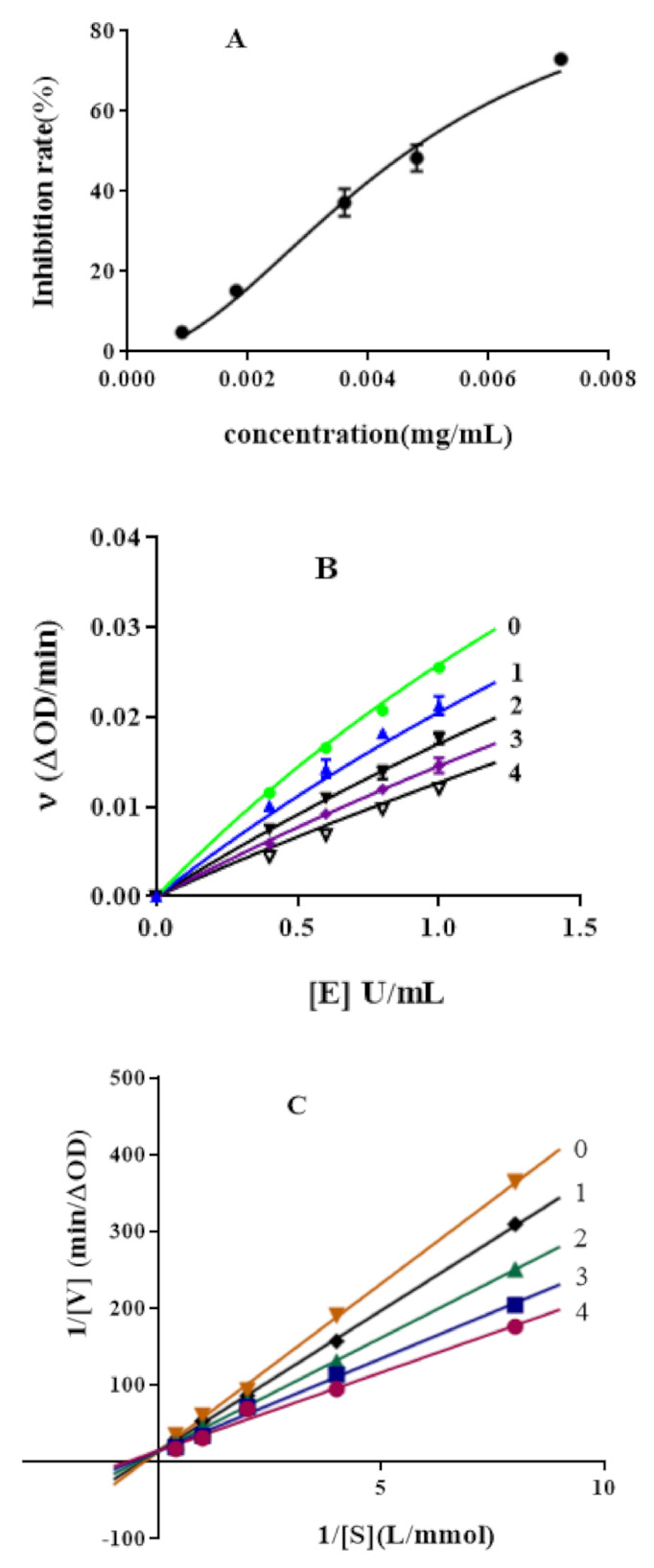

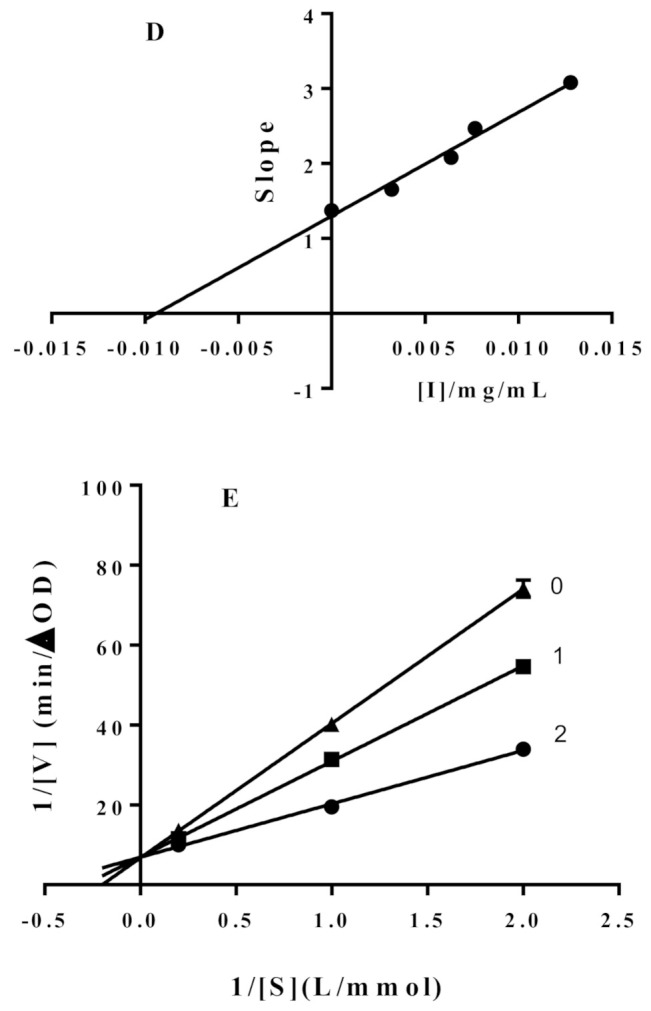
Inhibitory effect of hypericin on *α*-glucosidase activity (**A**); inhibitory mechanism of hypericin on *α*-glucosidase activity (**B**), hypericin concentrations in curves 0-4 were 0, 1.25, 2.5, 5, 10 mg/L, respectively; inhibitory type of hypericin on *α*-glucosidase activity (**C**), hypericin concentrations in curves 0-4 were 0, 1.25, 2.5, 5, 10 mg/L, respectively; inhibition constants (**D**) of hypericin on *α*-glucosidase; Inhibitory type of acarbose on *α*-glucosidase activity (**E**), acarbose concentrations on curves 0–2 were 0, 250, 500 mg/L, respectively.

The inhibition activity of hypericin against *α*-glucosidase showed a dose-dependent manner (Figure 1A). The IC_50_ of hypericin and acarbose was estimated to be 4.66 ± 0.27 mg/L and 863 ± 49 mg/L, respectively, which suggested that the inhibition potential of hypericin is superior to acarbose on *α*-glucosidase and significantly higher than sennoside A and B [33]. The number of hydroxyl groups on the benzene ring in the inhibitor structure has a certain effect on the binding of the inhibitor to *α*-glucosidase. There are six hydroxyl groups in the benzene ring of hypericin, while there are only two hydroxyl groups and one carboxyl group in the benzene ring of sennoside. This might lead to stronger interactions between hypericin and enzyme active sites [34].

### 2.2. Inhibitory Mechanism of Hypericin on α-Glucosidase Activity

The reversibility of hypericin’s inhibitory activity against *α*-glucosidase activity was evaluated using a plot of ν (reaction rate) versus [E] (concentration of *α*-glucosidase) for various inhibitor concentrations (Figure 1B). Figure 1B shows that all straight lines had passed through the origin, and the slope of the line decreased with increasing hypericin concentration. This meant that the existence of hypericin did not reduce the effective enzyme dosage while decrease *α*-glucosidase enzyme activity. These results suggested that the inhibitory effect of hypericin on α-glucosidase enzymes was reversible, and non-covalent interactions exist between *α*-glucosidase and hypericin [35].

### 2.3. Enzymatic Kinetics of α-Glucosidase Inhibition

According to the Lineweaver–Burk double inverted curve method, the inhibition kinetic curves of hypericin were applied to determine the inhibition type and inhibition constant, respectively. Figure 1C,E shows that when the concentration of hypericin and acarbose increased, the slope decreased, whereas the vertical axis intercept did not change. All the straight lines almost intersected the Y axis at one point, which indicated that hypericin and acarbose were competitive inhibitors of *α*-glucosidase [36]. A straight line was obtained by plotting the corresponding inhibitor concentration with the value of Michaelis constant (km’) under different concentrations of inhibitors. The inhibition constants (Ki) of hypericin and acarbose were 9.4 mg/L and 40.6 mg/L, respectively. Ki is the dissociation constant of the enzyme-inhibitor complex. The Ki of hypericin was lower than acarbose, which indicated that the inhibition of hypericin on *α*-glucosidase was stronger than that of acarbose [37].

### 2.4. SPR Analysis of the Interaction of Hypericin and α-Glucosidase

**Figure 2:**
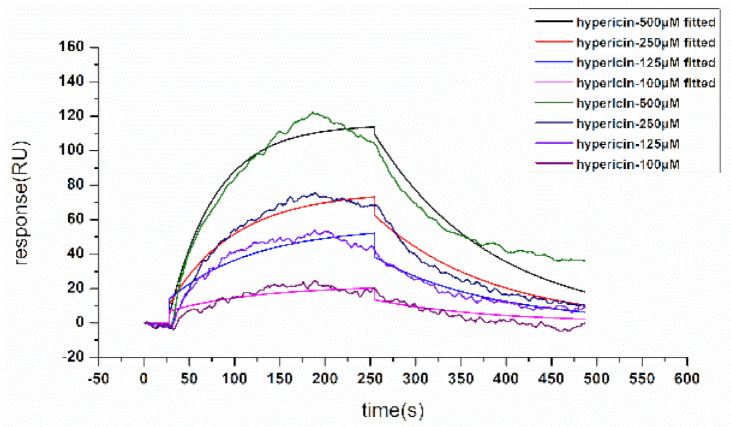
Surface plasmon resonance (SPR) concentration-signal graph.

Surface plasmon resonance (SPR) biosensors are powerful tools for the analysis of molecular interactions, and the association and disassociation of molecules can be assessed in real time without labels [38,39]. SPR biosensors have been widely used to study the biospecific interaction of low-molecular-weight compounds [40,41]. To assess the binding affinities between hypericin and *α*-glucosidase, we analyzed the binding and dissociation of hypericin (25-200 μM) with *α*-glucosidase that covered the chip surface. The response signal of the SPR biosensor generated kinetic information on the amount of complex that had formed on the chip surface at various hypericin compound concentrations. We injected PBS into the system periodically throughout each experiment; PBS served as the baseline reference to determine any systematic drifts over time. These findings indicate that hypericin can effectively bind to *α*-glucosidase to form a complex. The binding time of hypericin and *α*-glucosidase was 240 s, and the time of natural dissociation was 180 s (Figure 2). Association rate constant value and dissociation rate constant value, the main binding parameters for hypericin and *α*-glucosidase, were 118 mol^−1^·L·s^−1^, 0.00774 s^−1^, and KD 6.56 × 10^−5^ M, respectively, indicating that hypericin and *α*-glucosidase underwent the fast-binding and slow dissociation reaction, as well as strong binding.

### 2.5. Molecular Docking

Molecular docking is a theoretical simulation method to study the interaction between molecules and predict their binding modes and affinity such as ligands and receptors. To further understand the binding between hypericin and *α*-glucosidase, molecular docking was conducted in this study. The sequence identity and similarity between *α*-glucosidase from baker’s yeast and isomaltase (PDB ID: 3A4A) from *Saccharomyces cerevisiae* were 73% and 85%, respectively. The high sequence homology suggested that a high-quality 3D structure of *α*-glucosidase can be expected in molecular docking [42].

**Figure 3:**
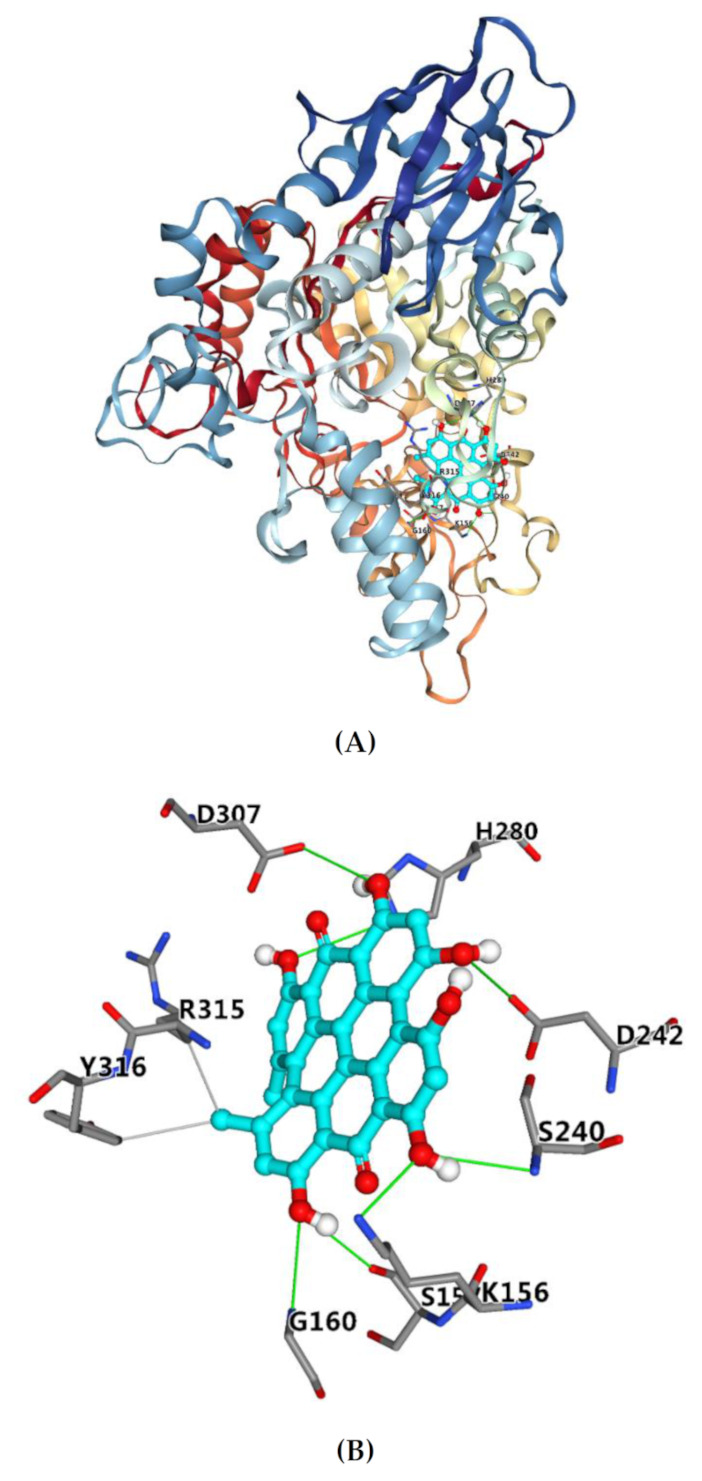
The molecular docking result of hypericin on *α*-glucosidase (**A**); the interaction of hypericin with the key residues in the active cavity of *α*-glucosidase (**B**).

Docking score analysis indicated that the target compound well fits into the active site, with a docking score of −8.4 kcal/mol. It has been reported that the binding energy of acarbose is −7.9 kcal/mol [43]. Therefore, the binding affinity of hypericin with *α*-glucosidase is stronger than that of acarbose with *α*-glucosidase. Figure 3A showed the binding pattern of hypericin with α-glucosidase. All of the binding sites were located on the active site of *α*-glucosidase. Therefore, hypericin is a competitive inhibitor of *α*-glucosidase, and the docking results are in line with the above inhibition kinetics.

Figure 3B shows that hydrophobic interactions (gray line) were formed by hypericin with amino acid residues Arg-315 and Tyr-316, and hydrogen bonds (green line) were formed between hypericin and amino acid residues Lys-156, Ser-157, Gly-160, Ser-240, His-280, Asp-242, and Asp-307. The main amino acid residues around acarbose were reported to include Tyr-158, Ser-240, Asp-242, Gln-279, Asp-307, Arg-315, Tyr-316, and Glu-411 [43], which suggested that hypericin and acarbose bind to *α*-glucosidase at the same or similar major amino acid residues, so the inhibition type of hypericin is comparable to that of acarbose. Some of these residues involved in the binding of hypericin to α-glucosidase were reported to play an important role in the catalytic mechanism of glycosidase [44]. Moreover, all seven hydrogen bonds formed between the phenolic hydroxyl group in hypericin and the amino acid residue with more than 2.01 Å bond length, respectively, which indicated that the hydrogen bond was an important force for hypericin to bind with glycosidase [45]. The formation of hydrogen bonds might reduce the hydrophilicity of α-glucosidase and increase its hydrophobicity, which was conducive to improving the stability of the complex [46]. In addition, the methyl of hypericin was inserted into the hydrophobic region to interact with residues Arg-315 and Tyr-316 which were reported to be located at the entrance of the active site pocket [47]. The phenomenon was consistent with the results from the inhibition kinetics analysis. Therefore, hypericin inserted into the active pocket of glucosidase prevented the substrate from entering, reduced the catalytic activity, led to the change of enzyme conception, and finally led to the inhibition of glucosidase activity. Yamamoto et al. reported that isomaltase contained three domains, namely A, B, and C, and domain A was shared by glycoside hydrolase family 13 [47]. The docking results showed the binding regions of hypericin with glucosidase were in domain A, which means the mechanism of hypericin binding to other enzymes of GH13 may be the same. Hence, the results of molecular docking may explain the observed high potential of hypericin as an *α*-glucosidase inhibitor.

## 3. Materials and Methods

### 3.1. Materials

*α*-glucosidase (EC 3.2.1.20) derived from *Saccharomyces cerevisiae* was obtained from Sigma-Aldrich (St. Louis, MO, USA) and dissolved in 0.1 M sodium phosphate buffer (pH 6.8). Hypericin (analytical grade) and acarbose were obtained from Shanghai Yuanye Bio-Technology Co., Ltd. (Shanghai, China). Stock solutions of acarbose and hypericin were prepared with dimethyl sulfoxide (DMSO). pNPG was obtained from Aladdin Reagent Co., Ltd. (Shanghai, China) and dissolved in 0.1 M sodium phosphate buffer (PBS pH 6.8). All other chemicals were of analytical grade. We used ultrapure water in all conducted experiments.

### 3.2. Determination of α-Glucosidase Activity Using Hypericin

The *α*-glucosidase inhibitory activity of hypericin was assessed as previously described [48,49]. Enzyme activity is represented by a change in reaction product concentration as catalyzed by glucosidase. In a 0.25-mL reaction system, the *α*-glucosidase solution (1 U/mL) was mixed with hypericin at different concentrations. The mixtures were then incubated at 37 °C for 10 min and later cooled to room temperature. The reaction was initiated through the addition of 50 μL of 0.5 mM pNPG (substrate) and incubated at 37 °C for 20 min. The reaction was later terminated by adding approximately 50 μL of 0.1 M Na_2_CO_3_. The absorption of the product at a wavelength of 405 nm was measured to determine the concentration by using an epoch2 spectrophotometer (BioTek Instruments, Inc., Winoosk, VT, USA). The enzymatic activity measured in the absence of an inhibitor was set as 100% and acarbose was employed as a positive control. We expressed the *α*-glucosidase inhibitory activity of hypericin using the median effective concentration for inhibitory activity (IC_50_), i.e., the amount of tested hypericin necessary to achieve a 50% decreased in *α*-glucosidase inhibitory activity. α-glucosidase enzyme inhibition rate was calculated using the following equation:

(1) $$\mathrm{Inhibition}\;\mathrm{rate}\left(\%\right)=\frac{Ac-\left(As-Asb\right)}{Ac}\times 100\%$$

where *Ac* is the absorbance value of control group, and *As* and *Asb* are the absorbance values of sample and background group, respectively.

### 3.3. Inhibitory Kinetic Analysis of α-Glucosidase Inhibition

To elucidate the inhibitory mechanism of hypericin on *α*-glucosidase, kinetic studies were conducted using Lineweaver–Burk and Dixon plots. The inhibitory effect of hypericin on *α*-glucosidase was assessed using the Lineweaver-Burk method [43]. The concentration of the enzyme was kept constant at 1 U/mL and different concentrations (0.125, 0.25, 0.5, 1, and 2.5 mM) of the reaction substrates (pNPG) were prepared. Various concentrations of hypericin (1.25–10 mg/L) were also provided. We plotted the double reciprocal lines of initial velocity (ν) against substrate concentration, which was then used to determine maximum reaction velocity (*Vmax*) and Michaelis constant (*Km*) using the Michaelis–Menten model. The Lineweaver–Burk equation was used to describe in a double reciprocal form the competitive inhibition mechanism [50]:

(2) $$\frac{1}{\nu }=\frac{Km}{Vmax}\left(1+\frac{\left[I\right]}{Ki}\right)\frac{1}{\left[S\right]}+\frac{1}{Vmax}$$

The secondary plot can be established from:

(3) $$\mathrm{Slope}=\frac{Km}{Ki}\left[I\right]+Km$$

where *ν* is the enzyme reaction velocity in the absence and presence of hypericin. *Km* and *Ki* represent the Michaelis-Menten constant and inhibition constant, respectively. The concentrations of hypericin and pNPG are indicated by [*I*] and [*S*], respectively. The secondary plot of slope against [*I*] is linearly fitted, assuming a single class of inhibitory sites or a single inhibitory site [48].

### 3.4. Hypericin and α-Glucosidase Interactions by SPR Measurements

The interaction between hypericin and *α*-glucosidase at room temperature was analyzed in real time on an OpenSPR instrument (Nicoya, Kitchener, Ontario, Canada). The COOH sensor chip was installed on the OpenSPR instrument as per standard procedures. After achieving a stable baseline, 10 mM HCl was injected to clean the surface of the chip and run for 1 min. The buffer flow rate was decreased to 20 μL/min, followed by injecting 200 μL of EDC/NHS (0.4 M/0.1 M) solution, which was freshly prepared, into the flow cell of the SPR instrument to activate the carboxyl situated on the chip surface. Then, 200 μL of *α*-glucosidase solution (0.5 mg/mL) diluted with sodium acetate (10 mM, pH 3.5) was injected into the flow cell to attach *α*-glucosidase onto the chip surface after deactivation of excess reactive groups on the surface by a 7-min liquid pulse of 200 μL blocking solution (20 μL/min, 4 min). Finally, different concentrations of hypericin (25–200 μM) were injected onto the surface of the *α*-glucosidase chip, and the sample was loaded at a rate of 20 L/min. The bound hypericin compound was then desorbed from the *α*-glucosidase coating surface using acid and alkali. After these measurements, the surface of the chip was then regenerated using a 100-μL injection of 2 mM NaOH. The localized surface plasmon resonance (LSPR) response changed over time and showed the dynamics of the chip surface binding events. The experimental results and dynamics were analyzed using TraceDrawer (Ridgeview Instruments AB, Uppsala, Sweden) and a one-to-one analysis model.

### 3.5. Molecular Docking Analysis

Molecular docking was performed to determine the interaction kinetics [51]. 3D structures of yeast α- glucosidase (EC 3.2.1.20) are unavailable. However, there are X-ray structures available for the isozyme isomaltase/a methylglucosidase (EC 3.2.1.10). The isomaltase with the PDB entry 3A4A has a high-resolution X-ray structure and high sequence homology (73%) [42]. Therefore, the molecular docking analysis was carried out by 3A4A that was obtained from Protein Data Bank (https://www.rcsb.org/structure/3A4A (accessed on 10 June 2021)) [43]. Hydrogen atoms were added onto the protein, and all water molecules were deleted using the AutoDock tools. The 3D structures of hypericin and acarbose were built in ChemBio 3D Ultra 19.0 and processed by adding hydrogen atoms, charges, and energy minimization. The grid box was set to contain the whole molecule. The docked model with the lowest docking energy was selected to represent its most favorable binding pattern, with prediction performed using AutoDock Vina [52].

### 3.6. Statistical Analysis

All experimental results including *α*-glucosidase activity assay and inhibitory kinetic analysis were measured thrice. The data were expressed as the mean value ± standard deviation (*n* = 3). Data analysis was performed with GraphPad Prism 7.

## 4. Conclusions

Using *α*-glucosidase inhibition experiments and enzyme kinetics study, we have shown that hypericin imparts a strong inhibitory effect on *α*-glucosidase, and this activity is reversible and involves competitive inhibition of *α*-glucosidase. This indicates that the binding sites of hypericin and the enzyme are within the active site. As the first-line drugs of type 2 diabetes, acarbose and voglibose are both competitive inhibitors of *α*-glucosidase. In the computational simulation study, the possible binding mode of hypericin and *α*-glucosidase was established, which was the same as the previous study on polyhydroxy compounds and played a very important role in all binding modes of hydrogen bond interaction [53]. The major amino acid residues of hypericin and acarbose binding to *α*-glucosidase were the same or similar. The binding properties of hypericin onto *α*-glucosidase were assessed using a label-free SPR detection system. The results revealed that hypericin binded to *α*-glucosidase and formed a new stable complex that exhibited the fast-binding and slow dissociation reaction, as well as strong binding.

In conclusion, the interaction between hypericin and *α*-glucosidase was studied by enzyme dynamics and computational simulation, and the possible binding mechanism was elucidated. Hypericin has a strong ability of enzyme inhibition, which is similar to acarbose in the aspects of inhibition type and binding mode. Therefore, hypericin may be potentially used as a natural substitute for an *α*-glucosidase inhibitor. This is the first study that describes the inhibitory activity of hypericin, which may potentially be applied to the food and industry medical field. In addition, further studies are needed, including those on the structure–activity relationship of hypericin to *α*-glucosidase inhibition and its consequences in vivo.

## Data Availability

Data is contained within the article.

## Abbreviations

AGI, alpha glucosidase inhibitors; DM, diabetes mellitus; DMSO, dimethyl sulfoxide; EDC, 1-ethyl-3-(3-dimethylaminopropyl)carbodiimide; IC_50_, maximum half inhibitory concentration; KD, balances dissociation constant; Ki, inhibition constant; Km, michaelis constant; LSPR, localized surface plasmon resonance; NHS, N-Hydroxy succinimide; PBS, phosphate buffer; pNPG, p-nitrophenyl-α-D-glucopyranoside; SPR, surface plasmon resonance; Vmax, maximum reaction velocity.
